# Understanding risks and consequences of pathogen infections on the physiological performance of outmigrating Chinook salmon

**DOI:** 10.1093/conphys/coab102

**Published:** 2022-01-21

**Authors:** F Mauduit, A Segarra, M Mandic, A E Todgham, M R Baerwald, A D Schreier, N A Fangue, R E Connon

**Affiliations:** 1 Department of Anatomy, Physiology & Cell Biology, University of California Davis, 95616 Davis, CA, USA; 2 Department of Animal Science, University of California Davis, 95616 Davis, CA, USA; 3 California Department of Water Resources, Division of Environmental Services, 95814 Sacramento, CA, USA; 4 Department of Wildlife, Fish, and Conservation Biology, University of California Davis, 95616 Davis, CA, USA

## Abstract

The greatest concentration of at-risk anadromous salmonids is found in California (USA)—the populations that have been negatively impacted by the degradation of freshwater ecosystems. While climate-driven environmental changes threaten salmonids directly, they also change the life cycle dynamics and geographic distribution of pathogens, their resulting host-pathogen interactions and potential for disease progression. Recent studies have established the correlation between pathogen detection and salmonid smolt mortality during their migration to the ocean. The objective of the present study was to screen for up to 47 pathogens in juvenile Chinook salmon (*Oncorhynchus tshawytscha*) that were held in cages at two key sites of the Sacramento River (CA, USA) and measure potential consequences on fish health. To do so, we used a combination of transcriptomic analysis, enzymatic assays for energy metabolism and hypoxia and thermal tolerance measures. Results revealed that fish were infected by two myxozoan parasites: *Ceratonova shasta* and *Parvicapsula minibicornis* within a 2-week deployment. Compared to the control fish maintained in our rearing facility, infected fish displayed reduced body mass, depleted hepatic glycogen stores and differential regulation of genes involved in the immune and general stress responses. This suggests that infected fish would have lower chances of migration success. In contrast, hypoxia and upper thermal tolerances were not affected by infection, suggesting that infection did not impair their capacity to cope with acute abiotic stressors tested in this study. An evaluation of long-term consequences of the observed reduced body mass and hepatic glycogen depletion is needed to establish a causal relationship between salmon parasitic infection and their migration success. This study highlights that to assess the potential sublethal effects of a stressor, or to determine a suitable management action for fish, studies need to consider a combination of endpoints from the molecular to the organismal level.

## Teaser

We compared health outcomes among juvenile Chinook salmon held in Sacramento River field sites to laboratory controls. All field fish acquired pathogens and lost energy in the form of body mass and liver glycogen. These findings highlight the ubiquity of pathogens and improve our understanding of mortality mechanisms for at-risk salmon populations.

## Introduction

Pacific salmon are iconic fishes that provide great economic, cultural and social benefit to humans (Riddell, 1993). They are considered keystone species, due in part to the abundance of nutrients they provide to both terrestrial and aquatic ecosystems (Cederholm *et al.,* 1999). Pacific salmon hatch in freshwater lakes and streams, migrate to the ocean during their first year of life, spend most of their ocean life in the ocean (generally 3–4 years) and eventually return to their natal river (a few days or weeks before spawning) (Groot, 1991). Salmonid natal homing behaviour and their frequently low dispersal between habitat patches results in a handful of species producing hundreds of genetically distinct runs, all with life histories adapted to local habitats (Moyle, 2002; Eliason *et al.*, 2011).

Despite their remarkable capacity for adaptation, salmonids are in severe decline in many of their native habitats (Montgomery, 2003). The greatest concentration of at-risk Pacific salmonid populations is in CA, USA, where 14 of 21 anadromous salmonid evolutionary significant units are State and federally listed as threatened or endangered and are anticipated to be extinct within the next century (Moyle *et al.,* 2017). Reasons for these declines are complex and multiple, including barriers to fish passage, historic over-fishing, predation by invasive species, habitat destruction, increased fluctuation in ocean temperature, degradation of water quality, hypoxia, increased temperature and disease (Lehman *et al.,* 2020).

Pathogens are regarded as a major cause of mortality in juvenile salmonids that migrate through the Pacific Northwest of North America (Foott *et al.,* 2002; Fujiwara *et al.,* 2011; Foott *et al.,* 2017), and resulting infections have been correlated with salmonid smolt mortality during their migration to the ocean (Hostetter *et al.,* 2011; Connon *et al.,* 2012; Miller *et al.,* 2014; Lehman *et al.,* 2020). Few studies, however, have established a causal relationship between co-infection occurrence and fish mortality. Salmonids are exposed to a wide variety of bacteria, protozoa, viruses and parasites throughout their lives (Buchanan *et al.,* 1983). Infections and resulting diseases are, however, of growing concern as water temperatures rise (Ray *et al.,* 2012). High river temperature generally enables the increase of pathogen replication (Ewing *et al.,* 1986; Crossin *et al.,* 2008; Ray *et al.,* 2012; Foott *et al.,* 2017) or infectious dose in the environment (Stocking *et al.,* 2006). California is the southernmost range extent for six anadromous salmonid species including endangered endemic populations of Chinook salmon (*O. tshawytscha*). These population are more frequently pushed towards their upper thermal limit compared to those from northern region (Zillig *et al.,* 2021), which potentially reduces fish resilience and resistance to infections (Mateus *et al.,* 2017). For outmigating juveniles, transformation from parr to smolt, and transition from freshwater to seawater, has also been shown to lead to reduced immunological protection, predisposing them to disease (Hoar *et al.,* 1997; Eggset *et al.,* 1999; Johansen *et al.,* 2016). Furthermore, studies have reported that infected sockeye salmon (*Oncorhynchus nerka*) can experience increased predation during their outmigration (Furey *et al.,* 2021; Miller *et al.,* 2014), presumably due to reduced ability to detect and escape predators or as a result of increased prey conspicuousness (Mesa *et al.,* 1994).

Recent pathogen research on salmonids has focused on the development of a set of molecular markers associated with stress and immune responses (Miller *et al.,* 2009, 2011; Connon *et al.,* 2012; Jeffries *et al.,* 2014). This has led to the discovery of new viruses and potential transmission dynamic in salmonids (Teffer *et al.,* 2020; Mordecai *et al.,* 2021) and highlighted the role of pathogens in fish migration success (Jeffries *et al.,* 2014; Bass *et al.,* 2019). While transcriptomic tools are useful for conservation research (Connon *et al.,* 2018), they are not without limitations, which include a lack of a mechanistic basis for their interpretation, complicated response patterns in wild animals and unclear links to Darwinian fitness (McKenzie *et al.,* 2007; Dantzer *et al.,* 2014). However, combining transcriptomic tools with endpoints at higher levels of biological organization, such as biochemical enzymes underlying key reactions, whole organism energetics and metabolism or complex physiological traits, can allow for the establishment of a causal relationship between a change in habitat quality and an organism’s functional integrity (Cooke and Suski, 2008; Adams and Ham, 2011).

Body mass and energy stores are important drivers of survival in juvenile salmonids (reviewed in Sogard, 1997). Hatchery-released Chinook salmon survival to maturity has been linked to a larger size at ocean entry (Claiborne *et al.,* 2011; Woodson *et al.,* 2013). Also, glycogen reserve and individuals’ capacity for energy mobilization determine their ability to cope with prolonged hypoxia, elevated temperature or sustained swimming (Johnston and Goldspink, 1973; Nilsson and Östlund-Nilsson, 2008; Corey *et al.,* 2017).

In aquatic environments, water temperature and oxygenation can undergo wide and rapid fluctuations. Any alteration in the ability of organisms to cope with these fluctuations is likely to affect their fitness (Somero, 2005; Mandic *et al.,* 2009). Loss of equilibrium (LOE) during a progressive increase in temperature or decrease in oxygen indicates thermal and hypoxia tolerance, respectively (Lutterschmidt and Hutchison, 1997; Anttila *et al.,* 2013; Roze *et al.,* 2013; Claireaux and Chabot, 2016). These two traits are highly variable between individuals, temporally stable and are determinants of survival and growth under free-ranging conditions (Claireaux *et al.,* 2013; Mauduit *et al.,* 2016, 2019). The LOE during progressive temperature increase and hypoxia are thus considered as ecologically relevant biomarkers of an organism’s functional integrity (Beitinger and McCauley, 1990; Davoodi and Claireaux, 2007; Pörtner and Knust, 2007; Wang and Overgaard, 2007; Claireaux and Davoodi, 2010). These endpoints have been used in several studies to assess the effect of pathogens. For instance, Atlantic salmon (*Salmo salar*) infected with Piscine orthoreovirus had a reduced hypoxia tolerance and increased susceptibility to warming (Lund *et al.,* 2017). Similarly, brown trout infected with *Tetracapsuloides bryosalmonae*, a myxozoan endoparasite, had a reduced upper thermal tolerance (Bruneaux *et al.,* 2017).

The present study aimed to screen for and evaluate potential pathogens infections in juvenile Chinook salmon (*Oncorhynchus tshawytscha*) deployed at key sites along a major migration corridor. The primary objective was to determine the consequences of pathogenic infection on fish performance and fitness, using a combination of indicators at multiple levels of biological organization. We hypothesized that pathogenic infection would elicit immune responses detectable at the molecular level (gene expression), which would also result in reduced growth and energy reserves and reduced tolerance to thermal and hypoxic stress.

## Materials and methods

### Fish transport and acclimation

All fish care and protocols were reviewed and approved by the University of California Davis (UC Davis) Institutional Animal Care and Use Committee (protocol no. 21338). Juvenile fall-run Chinook salmon (*N* = 300; mass, 2.57 ± 0.02 g; total length, 7.03 ± 0.02 cm) were transported from the Mokelumne River Hatchery (California Department of Fish and Wildlife, Clements, CA, USA) to the UC Davis Center for Aquatic Biology and Aquaculture (CABA) in early March 2019. Fish were transported in river water in an aerated transport tank that maintained oxygen levels of >90% of air saturation. Once at CABA, fish were held in outdoor, 2000-l tanks, with well-water flow-through (3 l min^−1^). Well-water salinity was <0.5 practical salinity unit (PSU) and temperature followed diurnal and seasonal variation measured in the river prior to deployment. Fish were fed daily to satiation with pelleted salmon diet (Salmon Sink, 2 mm; Skretting, USA).

### Fish tagging

Two weeks after their arrival to the CABA research facility, all fish were individually tagged, allowing the tracking of individuals throughout the experiment. For this, fish were anesthetized (MS-222; 100 mg/l, buffered with sodium bicarbonate), measured for total length (TL; 1 mm) and wet weight (WW; 0.01 g), tagged and placed into a recovery tank. PIT tags (Biomark MiniHPT8™, 134.2 kHz, 8.4 × 1.4 mm, 0.02 g in air) were injected subcutaneously, parallel with the long axis of the body, between the dorsal fin and the head of the animal. A Biomark syringe implanter (MK165) and a 16-gauge needle (N165 needle; 6-mm insertion depth, 1.5-mm needle width) were used to implant the PIT tag. During tagging, fish were immobilized in a wet cradle and the tag number was recorded using a Biomark HPR Lite Reader. Chlorhexidine (2% v/v) was used to disinfect each individual’s skin prior to injections in order to prevent post-surgical infections. All equipment was also sanitized in a chlorhexidine 2% solution before tagging each fish. Fish recovery (99%) was closely monitored post-injection for a period of 15 days.

### Experimental protocol and study sites

**Figure 1:**
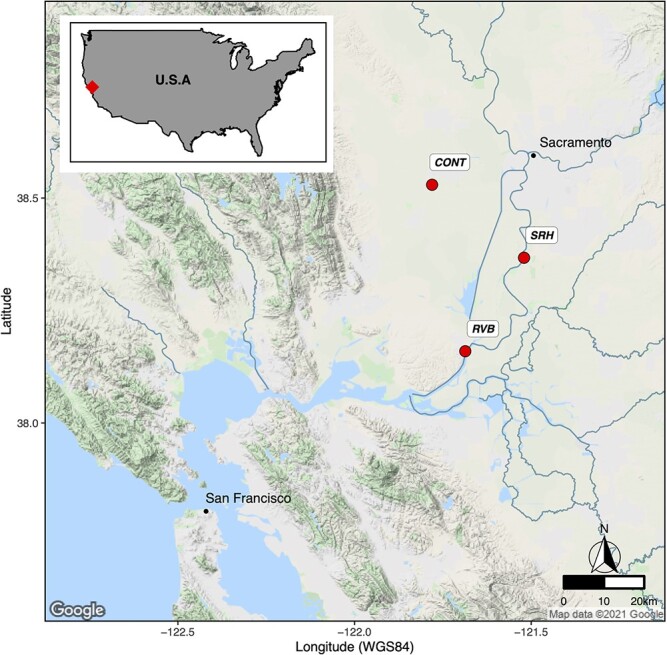
Fish deployment sites. Map coordinates are expressed in decimal degrees. CONT, control group maintained in the UC Davis rearing facility over the whole experiment; RVB, group deployed for 2 weeks into the Sacramento River at Rio Vista Bridge (38.1585°N, 121.6830°W); SRH, group deployed for 2 weeks into the Sacramento River at Hood (38.3677°N, 121.521°W).

Following a post-tagging period of 30 days, all fish were submitted to a first set of hypoxia and thermal tolerance tests (see 2.9) 2 weeks and 1 week prior to deployment (T0), respectively, at key locations in the Sacramento River (described below). These tests allowed for the even distribution of fish into three groups of similar hypoxia and upper temperature tolerance. The control group (CONT) was kept at the CABA research facility throughout the experiment under temperature conditions matching the field deployments, and fish were held in cages during the deployment period. The other two groups were transported and deployed in cages (diameter, 45 cm; height, 80 cm; *N* = 3 cages; 33 fish per cage) for 14 days in the Sacramento River at Rio Vista, CA, USA (RVB) and Hood, CA, USA (SRH), respectively (Fig. 1). Fish wet weight was measured 1 day before deployment and at the end of the field period, before bringing the fish back to the CABA research facility.

The two sites of interest (RVB and SRH) are 32 km apart and have continuous water quality monitoring stations, which are managed by the California Department of Water Resources’ Division of Operations and Maintenance (cdec.water.ca.gov/), allowing for the monitoring of water parameters such as temperature, dissolved oxygen concentration, salinity and flow in real time. Cages were deployed at the monitoring sites, suspended from the water surface using flotation devices. RVB and SRH are considered as integrator sites of the San Francisco Bay Delta (SFBD). SRH captures waters downstream of the discharge point from the wastewater treatment plant into the Sacramento River, south of the city of Sacramento. RVB is an informative site in terms of the Sacramento River flow. Flow magnitude has been reported as a driver determining the survival of Chinook salmon through the SFBD, with higher survival rates associated with higher flows (Brandes and McLain, 2001). The Sacramento River flows are regulated for salmonid passage through the SFBD based on data collected at RVB (NMFS, 2009). RVB is also a critical location on the salmon outmigration route, as it is located downstream of the Cache Slough Complex, a nursery area for outmigrating salmonids. RVB is under tidal influence with a daily flow reversal. SRH and RVB are not subjected to seawater intrusion, and salinity typically remains at <1 PSU.

### Tissue collection

Nine fish per group were sacrificed before fish deployment and six fish per group (two per cage) in CONT and RVB groups and seven fish in the group SRH were sampled at 14 d. Individuals were euthanized using an overdose of buffered tricaine methanesulfonate (MS-222, 500 mg/l), weighed (g) and measured (TL, mm) and sampled for the whole left gill, liver and heart. All tissues were immediately frozen in liquid nitrogen and stored at −80°C for subsequent analyses in our laboratory.

**Table 1:** Summary of the 47 infectious agents of interest

Agent type	Species	Abbreviation
Bacterium	*Aeromonas hydrophila*	ae_hyd
Bacterium	*Aeromonas salmonicida*	ae_sal
Virus	*Atlantic salmon paramyxovirus*	aspv
Bacterium	*Candidatus Branchiomonas cysticola*	c_b_cys
Parasite	*Ceratomyxa shasta*	ce_sha
Parasite	*Cryptobia salmositica*	cr_sal
Parasite	*Dermocystidium salmonis*	de_sal
Parasite	*Facilispora margolisi*	fa_mar
Bacterium	*Flavobacterium psychrophilum*	fl_psy
Virus	*Flavivirus salmonis*	fl_sal
Parasite	*Gyrodactylus salaris*	gy_sal
Parasite	*Ichthyophonus hoferi*	ic_hof
Parasite	*Ichthyophthirius multifiliis*	ic_mul
Virus	*Infectious hematopoietic necrosis virus*	ihnv
Virus	*Infectious salmon anemia virus*	isav8
Parasite	*Kudoa thyrsites*	ku_thy
Parasite	*Loma Salmonae*	lo_sal
Bacterium	*Moritella viscosa*	mo_vis
Parasite	*Myxobolus articus*	my_arc
Parasite	*Myxobolus cerebralis*	my_cer
Parasite	*Myxobolus insidiosus*	my_ins
Fluke	*Nanophyetus salmincola*	na_sal
Parasite	*Neoparamoeba perurans*	ne_per
Parasite	*Nucleospora salmonis*	nu_sal
Virus	*Salmonid herpesvirus/Oncorhynchus masou herpes virus*	omv
Parasite	*Parvicapsula kabatai*	pa_kab
Parasite	*Parvicapsula minibicornis*	pa_min
Parasite	*Parvicapsula pseudobranchicola*	pa_pse
Parasite	*Paranucleospora theridion (syn. Desmozoon lepeophtherii)*	pa_ther
Bacterium	*Piscichlamydia salmonis*	pch_sal
Bacterium	*Piscirickettsia salmonis*	pisck_sal
Virus	*Piscine myocarditis virus*	pmcv1
Virus	*Piscine reovirus*	prv
Bacterium	*Renibacterium salmoninarum*	re_sal
Bacterium	*Rickettsia-like organism*	rlo
Virus	*Salmon alphavirus 1, 2 and 3*	sav
Bacterium	*Gill chlamydia*	sch
Virus	*Infectious salmon anemia virus*	snow7
Parasite	*Sphaerothecum destructuens*	sp_des
Parasite	*Spironucleus salmonicida*	sp_sal
Parasite	*Tetracapsuloides bryosalmonae*	te_bry
Bacterium	*Tenacibaculum maritimum*	te_mar
Virus	*Viral erythrocytic necrosis virus*	ven
Virus	*Viral encephalopathy and retinopathy virus*	ver
Bacterium	*Vibrio anguillarum*	vi_ang
Bacterium	*Vibrio salmonicida*	vi_sal
Bacterium	*Yersinia ruckeri*	ye_ruc

**Table 2:** List of the 25 candidate genes of interest and 3 housekeeping genes

Gene name	Abbreviation	Function
Cytochrome P450 Family 1 Subfamily A Member 1	CYP450	General stress
Glutathione-s-Transferase 3	GST3	General stress
Glutathione-s-Transferase alpha	GSTα	General stress
Heat Shock protein serpin H1	HSP47	General stress
Heat Shock Protein 90 kDa AA1-inducible form	HSP90α	General stress
Heat Shock Protein 90 kDa alpha Beta 1	HSP90αβ	General stress
Ammonium transporter	RHCG	General stress
Glyceraldehyde-3-Phosphate Dehydrogenase	GAPDH	Housekeeping gene
60S Ribosomal gene 7 l	RPL7	Housekeeping gene
Ribosomal Protein S9	RPS9	Housekeeping gene
Beta-2-Microglobulin	B2M	Immune system
C-type Lysozyme	C-Lys	Immune system
Complement factor CF3	CF3	Immune system
Complement factor BF-2	CFB	Immune system
Cold inducible RNA Binding Protein	CIRBP	Immune system
Chemokine Receptors 5	CR5	Immune system
Chemokine Receptors 6	CR6	Immune system
Chemokine Receptors 7	CR7	Immune system
Classical Immunoglobulin	IgM	Immune system
Chemokine Interleukin 1 beta	IL-1β	Immune system
Chemokine Interleukin 8	IL-8	Immune system
Major Histocompatibility complex II	MHC2	Immune system
MX protein	MXpro	Immune system
Serum amyloid protein A	SAA	Immune system
T-cell receptor alpha	TCRα	Immune system
T-cell receptor beta	TCRβ	Immune system
Toll like receptors 1	TLR1	Immune system
Toll like receptors 2	TLR2	Immune system
Toll like receptors 3	TLR3	Immune system
Tumour necrosis factor alpha	TNF-α	Immune system

### RNA extraction and cDNA synthesis

Gill tissues were homogenized using a Qiagen TissueLyser LT (Qiagen) and RNA was extracted using the RNeasy Plus Mini kit with gDNA eliminator columns (Qiagen). All extractions were performed with a QIAcube instrument (Qiagen, QIAcube System 230 V) according to the manufacturer’s protocol. RNA concentration was assessed by measuring the A260:280 and A260:230 ratios on a NanoDrop (ND1000 Spectrophotometer, NanoDrop Technologies, Inc., Wilmington, DE, USA) and quality checked by electrophoresis. RNA (500 ng) was reverse transcribed to cDNA using QuantiNova Reverse Transcriptase kit (Qiagen) with integrated genomic DNA removal as per the manufacturer’s protocol. All cDNA samples were diluted 1:4 with RNAse free water.

### Pathogen detection and host gene expression

Gill cDNA samples were individually analysed to detect the presence of 47 pathogens (Table 1) and to study gene expression patterns for 25 salmon genes (Table 2) using a microfluidics qRT-PCR platform (96.96 Dynamic ArrayTM IFC, Fluidigm BioMarkTM, Fluidigm Corp., San Francisco, CA, USA). This 96.96 dynamic arrayTM has similarly been used to assess salmon pathogen load or analyse immune response genes in salmon (Jeffries *et al.,* 2014; Miller *et al.,* 2016). In our study, each cDNA sample was run in duplicate with probes to detect 11 viruses, 14 bacteria and 21 parasites, known or suspected to cause tissue degradation or disease in salmonids worldwide (Miller *et al.,* 2016; Soto *et al.*, 2020) or in triplicate to study the general stress and immune responses. As the BioMark microfluidics platform uses very small assay volumes (7 nl), a cDNA pre-amplification step is required to optimize sensitivity of detection. Therefore, 1.25 μl of cDNA from each sample was pre-amplified with primer pairs corresponding to pathogen (Supplemental Data S1) or host genes (Supplemental Data S2) respectively, in a 5-μl reaction volume using Preamp Master Mix (Fluidigm). ExoSAP-IT (Affymetrix) was used to remove unincorporated primers and then pre-amplified samples were diluted 1:5 in Milli-Q water. Then, pathogen detection and relative gene expression were run on 96.96 dynamic array plates, using samples from the same pool of cDNA. Details of qPCR protocol on the BioMark platform are described in Miller *et al*. (2014, 2016). Briefly, 5 μl aliquots of sample premix (1x TaqManTM Gene Expression Master mix (Applied Biosystems™), 1x GE Sample Loading Reagent (Fluidigm) and 2.25 μl pre-amplified cDNA) were loaded into the wells corresponding to samples inlets from the 96.96 dynamic arrayTM (Fluidigm). Then, 5 μl of the assay premix (1x assay loading reagent (Fluidigm), 9 μM of each primer and 2 μM probe) were loaded into the assay inlets wells from the same 96.96 Fluidigm plate. The chip was mixed in an IFC controller HX (Fluidigm) and placed in the Fluidigm BiomarkTM HD system where PCR was performed under the following conditions: 50°C for 2 min, 95°C for 10 min, followed by 40 cycles of 95°C for 15 s and 60°C for 1 min. Pathogen quantification (load of pathogen) was calculated using five serial dilutions of synthesized DNA strings (positive controls) corresponding to each pathogen target (Miller *et al.,* 2016), then results were computed as log_10_ (RNA copy number). As described by Miller *et al.,* 2016, RNA assays were conducted to detect pathogens that were in an active state at the time of sampling. This, however, can distort the pathogen number calculation since mRNA expression level can be highly variable. As such, copy number was used as method to reflect relative abundance among samples and should not be extrapolated as genome copy numbers between samples (Miller *et al.,* 2016). Concerning the fish gene expression, relative mRNA levels were calculated using the comparative Ct method (Livak and Schmittgen, 2001). Gene expression data were normalized to the three housekeeping genes (Glyceraldehyde-3-phosphate dehydrogenase, Ribosomal Protein S9, 60S Ribosomal Protein L7; Table 2). No significant differences in Ct values were observed for these three genes between treatments (one-way ANOVA, *P* > 0.05; coefficients of variation, 6.0%, 4.0% and 7.0%, respectively).

### Metabolite extraction, glucose and glycogen assays

Each liver was weighed and transferred to 2-ml tubes and immediately sonicated on ice in 500 μl of 8% perchloric acid using four 6-s bursts of a Micro Ultrasonic Cell Disrupter (Model 120 Sonic Dismembrator, Fisher Scientific) set on its highest setting. A 100-μl aliquot of the homogenate was frozen at −80°C for subsequent glycogen digestion. The remaining homogenate was centrifuged at 10000 g for 10 min at 4°C, and the resulting supernatant was neutralized with 40 μl 3 M K_2_CO_3_. Neutralized extracts were further centrifuged a second time to remove any further precipitates and stored at −80°C until later analysis. Extracts were then thawed on ice and assayed spectrophotometrically (BioTek, Synergy HTX) for glucose at 340 nm according to protocols outlined in Bergmeyer (1983). Specifically, 20 μl of sample or 20 μl of glucose standard was pipetted into a 96-well plate, followed by 100 μl of assay buffer. The plate was incubated at 37°C for 5 min, and after the initial absorbance was read, 2.25 U/ml of hexokinase was added in each well and the plate was incubated at 37°C for 20 min prior to final absorbance read.

The 100-μl homogenate set aside for glycogen analysis was thawed on ice and digested to glucose according to protocols outlined in Bergmeyer (1983). The thawed samples were partially neutralized with 15 μl 3 M K_2_CO_3_ and buffered in 500 μl 0.45 M sodium acetate (pH 4.8). To digest glycogen to glucose, 10 μl of amyloglucosidase was added to each sample and samples were incubated in a 37°C water bath for 2 h. The enzymatic reaction was stopped by adding 10 μl of 70% HClO_4_ and samples were placed on ice for 10 min. Then, 30 μl of 3 M K_2_CO_3_ was added to each sample, samples were centrifuged at 1000 *g* for 10 min at 4°C and assayed for glucose as described above. Total glycogen content was calculated by subtracting free glucose measured in neutralized extracts from glucose obtained in digested samples. Glycogen was expressed as micromoles of glycosyl units per gramme of wet weight.

### Enzyme activity assays

Each heart was weighed and placed in 500 μl of ice-cold homogenizer buffer (50 mM HEPES, 5 mM dipotassium ethylenediaminetetraacetic acid, 0.1% Triton X-100, pH 7.4) and immediately sonicated on ice using four 6-s bursts set on the highest speed setting. The homogenate was centrifuged at 1000 *g* for 2 min at 4°C, and the resulting supernatant was divided in separate aliquots for the analysis of lactate dehydrogenase (LDH), pyruvate kinase (PK) and citrate synthase (CS) enzyme activity. Maximal enzyme activities were determined spectrophotometrically (BioTek, Synergy HTX) by measuring the accumulation or disappearance of nicotinamide adenine dinucleotide (NADH) at 340 nm (LDH, PK) and the appearance of 5-thio-2-nitrobenzoic acid (TNB) as a result of the reaction of free CoA with 5,5′-dithiobis (2-nitrobenzoic acid) (DTNB) at 412 nm (CS) at 18°C, over a 10-min incubation period. The assay conditions were as follows (in mM): LDH = 50Tris, pH 7.4, 2.5 pyruvate, 0.6 NADH; PK = 50Tris, pH 7.4, 0.5 NADH, 5 ADP, 0.01 fructose-1,6-biphosphate, 100 KCl, 10 MgCl2, 5 phosphoenolpyruvate and 50 U/ml LDH; and Tris, pH 8.0, 0.5 oxaloacetate, 0.3 acetyl-CoA, 0.15 5,5-dithiobis-2-nitrobenzoic acid. Substrates such as pyruvate (LDH), phosphoenolpyruvate (PK) and oxaloacetate (CS) were at saturating levels and were omitted in control reaction rates in order to calculate background reaction rates. We used empirically determined extinction coefficients to quantify maximal activity for each enzyme. Total soluble protein was determined in each homogenate using the Bradford protein assay (Sigma-Aldrich; Bradford, 1976). Maximal enzyme activities were normalized to tissue weight and total soluble protein.

### Challenge tests

The hypoxia and temperature challenge tests (HCT and TCT, respectively) were conducted as described in Mauduit *et al.* (2016, 2019) to assess hypoxia and upper temperature tolerance. Challenges were performed 2 weeks (HCT #1) and 1 week (TCT #1) before field deployment and 1 week (HCT #2) and 2 weeks (TCT #2) after the field exposure. Hypoxia challenges were always performed first, and the two challenge tests were both conducted on the same fish, 1 week apart, to allow fish time to recover from the challenge tests and reduce the effects of repeated handling.

Hypoxia challenge test (HCT)

The hypoxia challenge test consisted of an initial, rapid decrease in water oxygenation from nearly 100% to 20% air saturation over 1 h, followed by a slower decrease in oxygenation at 2% air saturation/h until all fish reach LOE. Hypoxia was achieved by bubbling nitrogen gas through a submersible pump placed in the test tank. Water oxygenation was continuously monitored using an optical dissolved oxygen metre (ProSolo ODO, YSI). When LOE was achieved, each fish was quickly removed from the test tank, identified by RFID tag and placed in a fully aerated tank to recover. The time to LOE per fish and corresponding oxygen level were recorded. Time to LOE was measured from the start of the oxygen decline. All tested fish recovered.

Temperature challenge tests (TCT)

The temperature challenge tests consisted of an initial, rapid increase in water temperature, from acclimation temperature to 26°C, over 2.5 h, followed by a slower increase (0.5°C/h) until all the fish lost equilibrium. Water temperature was controlled using two 1700-W heaters (Process Technology, Smart One). Fish acclimation temperature was 10°C in March and 17°C in May (set to match hatchery and river temperatures, respectively). A submersible pump placed in the tank ensured homogeneity of water temperature and air saturation (controlled by bubbling of a mixture of oxygen and air in the tank). When a fish lost its ability to maintain balance, it was quickly removed from the rearing tank, identified by RFID tag and placed in a fully aerated tank at acclimation temperature to recover. The time to LOE and corresponding temperature were also recorded. All tested fish recovered.

### Data analyses

Pathogen detection and gene expression were analysed using a two-way ANOVA with the pathogens/genes and sites as factors. Growth during the field period, glucose/glycogen concentrations and enzyme activities were analysed using a one-way ANOVA with sites as factor followed by a Tukey’s multiple comparisons test. The assumptions of normality and homogeneity of variance were assessed using the Shapiro–Wilk and Levene’s tests. Deployed fish survival and responses to challenge tests were analysed using a Kaplan–Meier survival analysis, followed by a log-rank test with the Holm-Sidak method for multiple comparisons. A PCA was applied to the copy number of pathogens detected, differentially regulated genes, hepatic glycogen concentration and growth in the individuals sampled at the end of the field period using FactoMineR and factoextra R packages. This analysis aimed to describe the interactions between fish pathogen load and changes in health indicators, as well as identifying the main drivers differentiating the treatment groups. Figures were created and statistical analyses were performed with R (4.0.2) and Prism 8. Results were declared statistically significant at the two-tailed 5% (i.e. *P* < 0.05). Throughout the manuscript results are presented as mean ± standard error.

## Results

### Field caging

**Figure 2:**
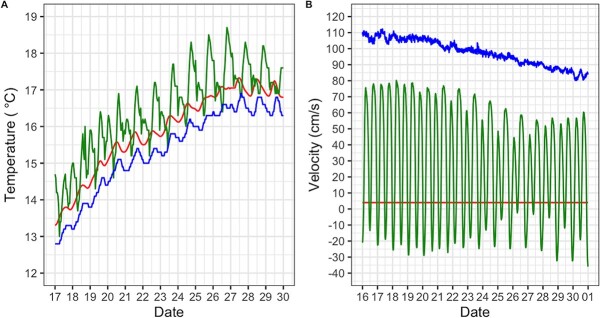
Water temperature (°C, **A**) and flow velocity (cm.s^−1^, **B**) measured during the 2 weeks of field exposure. Red line, fish from the CONT group; green line, fish deployed in the RVB; blue line, fish deployed in the SRH. Data at deployment sites were measured by continuous monitoring stations (nwis.waterdata.usgs.gov) and using a temperature logger (Hobo Pendant 64 K) and a handheld flow velocity meter in the control (Hoentzsch HFA-Ex).

During the 2-week field period, temperature at RVB varied from 13 to 18.7°C with daily fluctuations ranging from 1.5 to 2.5°C (Fig. 2). Temperatures at SRH were cooler and more stable than at RVB, with a minimum recorded temperature of 12.8°C and a maximum of 16.9°C and daily fluctuations of <1°C. Mean daily temperature was lower at SRH than at RVB by 1.3 ± 0.3°C, concomitant with the higher flow observed at SRH than at RVB (97.7 ± 0.2 vs 36.5 ± 0.6 cm.s^−1^). CONT temperature also followed seasonal variation and ranged from 13.3°C at the beginning of the deployment period to 17.3°C towards the end, with daily fluctuation of <1°C. Moreover, tidal influence induced daily flow fluctuations of up to 80 cm.s^−1^ at RVB while none were observed at SRH. It must be noted these values of flow velocity are calculated from the total river discharge and do not necessarily represent the flow the fish experienced. Flow in the cages was presumably lower than the river mean, due to the mesh impeding the current and progressively clogging because of organic matter accumulation.

**Figure 3:**
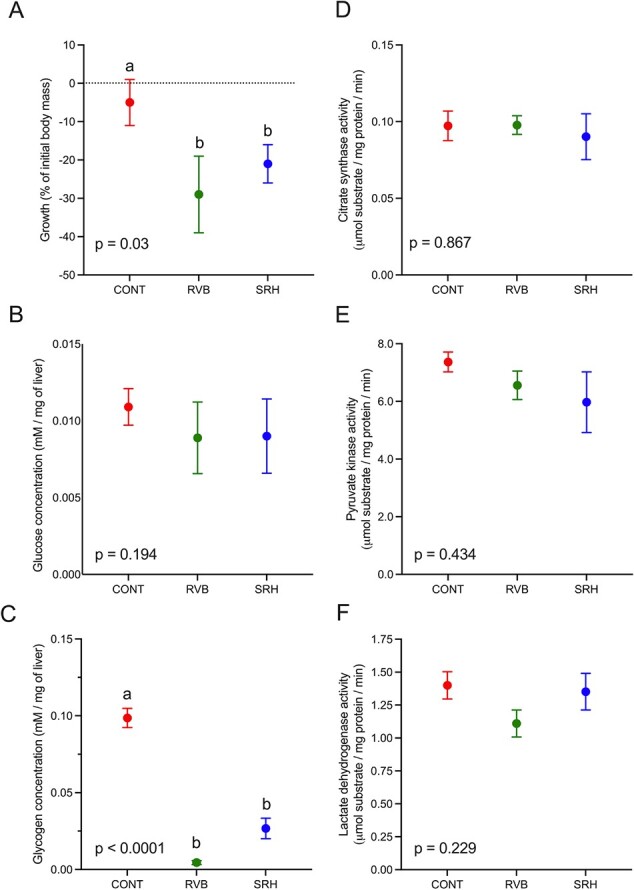
Energetic metabolism. (**A**) Growth in % of initial body mass calculated from mass measured one day before field deployment and at the end of the 2 weeks field period in the fish from the CONT group and in the fish deployed in the RVB and SRH. Glucose (**B**) and glycogen (**C**) concentrations in μmol/g of wet liver and cardiac activity of the CS (**D**), PK (**E**) and LDH (**F**) in mmol substrate/mg protein/min from fish heart sampled at the end of the field exposure. *N* = 6–7/group. Data are presented as mean +/− SEM. Letters indicate statistical significance (*P* < 0.05, one-way ANOVA followed by a Sidak’s multiple comparisons test).

Survival in the RVB and SRH groups was >90% with no significant difference between sites (Kaplan–Meier, *P* = 0.91). Fish that died in the field did not perform differently, prior to deployment, in the hypoxia and temperature challenge tests. Growth during the field period was calculated from fish mass measured one day before the fish deployment into the river and at collection at the end of the field period (Fig 3A). Body mass of fish from the control group held at the research facility did not change during the 2-week period of caging (−5 ± 6% of initial body mass). On the other hand, fish placed in cages at RVB and SRH displayed a significant reduction in body mass (−29 ± 10 and −21 ± 5% of initial body mass) compared to the control group, representing a decrease from 6.3 ± 0.5 g and 6.0 ± 0.4 g to 5.0 ± 0.5 and 4.9 ± 0.2 g in the RVB and SRH groups (*P* = 0.03), respectively.

### cDNA pathogen detection in the gills

Of the 19 juvenile Chinook salmon (6 CONT, 6 RVB and 7 SRH) screened for pathogens only the river-exposed fish tested positive for infections (Supplemental Data S3). Fish sampled prior to deployment (*N* = 9) did not test positive for any of the 47 agents. At the end of the field exposure, fish from the control group remained uninfected; however, all fish deployed in the Sacramento River (SRH or RVB) for 2 weeks tested positive only for the myxozoan parasites *Ceratonova shasta* (Ce_sha) and *Parvicapsula minibicornis* (Pa_min). No differences in pathogen DNA copy number/μl, and prevalence in fish gills was found between the different pathogens and river locations (two-way ANOVA, *P* > 0.1).

### Gene expression

**Figure 4:**
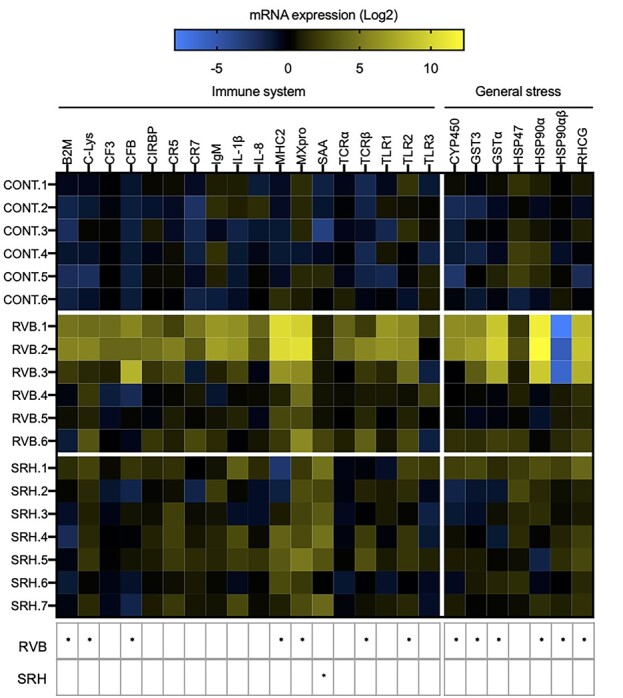
Heatmap illustrating the expression level of 25 genes involved in fish immune and cellular stress responses measured after the 2-week caging period in the RVB and SRH. CONT fish were held under similar conditions in the research facility. *N* = 6–7/group. Colours represent the mRNA expression levels. All transcripts were calibrated using samples collected before deployment (T0) and normalized with three housekeeping genes. Gene expression is presented relative to T0. Black colour indicates no change in expression, blue represents downregulated genes and yellow represents upregulated genes (Log2 fold-change). Each column corresponds to one of 25 candidate genes of interest (immune or cellular stress response) and each row represents an individual. Responses that were significantly different (two-way ANOVA Sidak’s multiple comparisons test) to the CONT group are summarized the table below the heatmap (^*^P < 0.05).

Of the 25 tested genes involved in the fish immune system or general stress response (Table 2), 14 were found to be influenced by field exposure (Fig. 4). When compared with the control, mRNA levels of 12 genes in gills were significantly up-regulated in the RVB group. Among these genes up-regulated (two-way ANOVA, *P* < 0.05), 7 were involved in immune response (*β2M*, *C-Lys*, *CFB*, *MHC2*, *MxPRO, TCRβ* and *TLR2)* and 5 in general stress response (*Cyp450*, *GST3*, *GSTα*, *HSP90α* and *RHCG*). The mRNA levels of one gene, *HSP90αβ*, which associated with the general stress response, were found to be down-regulated in the RVB group compared to the control. In the SRH group only one gene—involved in the immune response, *SAA*—was found significantly up-regulated compared to the control group (Fig. 4).

### Metabolite assays and enzyme activity

Glucose and glycogen concentrations were measured in fish livers at the end of the field exposure period across all three groups (*N* = 6–7/treatment; Fig. 3B and C, respectively). Hepatic glucose concentration in control fish was 5.08 ± 0.78 μmol/g of wet liver. There were no significant differences in hepatic glucose concentrations between any of the experimental groups (*P* = 0.194). Hepatic glycogen concentrations were significantly lower in the river-deployed fish (0.25 ± 0.15 and 3.1 ± 1.6 μmol/g of wet liver in the groups RVB and SRH, respectively) relative to the control group (CONT, 31.0 ± 4.7 μmol/g of wet liver, *P* > 0.0001). No difference was found between either river site (*P* = 0.739). PK, CS and LDH activities measured in fish hearts sampled from river-deployed fish were not significantly different from the control group (Fig. 3C–E; *P* = 0.867, 0.434 and 0.229 for PK, CS and LDH, respectively).

### Association between pathogen detection and sub-organismal biomarkers responses.

**Figure 5:**
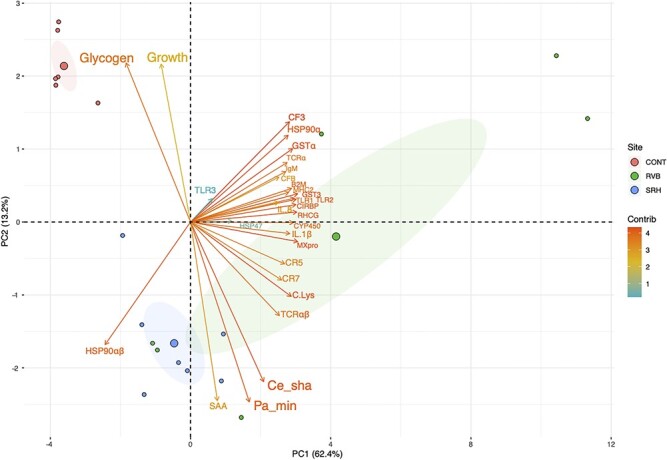
Biplot PCA describing the association between pathogens copy number detection, genes expression, hepatic glycogen concentration and body mass. Samples were collected at the end of the 2-week period of field exposure (*N* = 6–7/group). Red dots, fish from the CONT group; green dots, fish deployed in the RVB; blue dots, fish deployed in the SRH. The gradient of colour represents the total contribution of each variable (yellow, low contribution; red, high contribution). Arrow length approximates the variance of the variables. Arrows pointing in the same direction indicate that the corresponding variables are positively correlated.

PCA was applied to the copy number of pathogens detected, mRNA levels of differentially regulated genes, hepatic glycogen concentration and growth in the individuals sampled at the end of the deployment period (Fig. 5; *N* = 6–7/treatment). The two dimensions (PC1 and PC2) explained 75.6% of total variation. This PCA demonstrates that there were differential clusterings between fish that were deployed in the river and those from the control group. Fish from the group CONT were differentiated from the fish of the RVB group on PC1 (62.4%) and fish from the SRH group were partitioned from the fish of the CONT group on PC2 (13.2%). Arrows length and direction indicate that *MXpro, GST3, RHCG* and *CIRPB* were the most differentiating genes along PC1, not correlating with pathogen detection. Associated with PC2, *C. shasta* (Ce_sha) and *P. minibicornis* (Pa_min) infections positively correlated with SAA expression, which negatively correlated with growth and hepatic glycogen concentration.

### Hypoxia and temperature challenge tests

**Figure 6:**
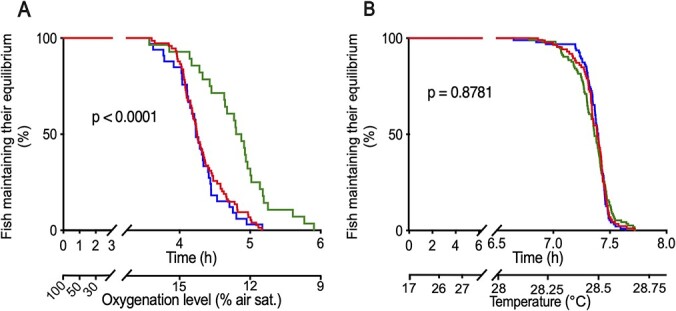
Fish response to hypoxia (**A**) and temperature (**B**) challenge tests conducted 1 and 2 weeks after their field deployment, respectively. Red line, fish from the CONT group (*N* = 93); green line, fish deployed in the RVB (*N* = 81); blue line, fish deployed in the SRH (*N* = 85). Corresponding oxygen level (**A**; % air sat.) and temperature (**B**; °C) are indicated on the secondary x-axis for information. *P*-values were computed using a Log-rank test.

Consequences of the 14-d river deployment to fish fitness were assessed through laboratory-based hypoxia and temperature challenge tests conducted 1 and 2 weeks after fish recovery from the field, respectively (Fig. 6). Mean time to LOE (TLOE) in the hypoxia challenge test for the fish from the control group was 4.33 ± 0.04 h, which corresponded to 13.7 ± 0.1% of air saturation (Fig. 6A). Fish from the SRH group displayed a similar hypoxia tolerance to the control fish (mean TLOE = 4.27 ± 0.06 h; *N* = 85); however, individuals from the RVB group had a higher hypoxia tolerance relative to the controls at the same time point (mean TLOE = 4.79 ± 0.09 h; 12.4 ± 0.2% air sat.; *N* = 81). The mean temperature challenge test TLOE (Fig. 6B) of the control group was 7.36 ± 0.01 h, which corresponded to a temperature of 28.5 ± 0.2°C (*N* = 93). Upper thermal tolerance of RVB and SRH groups were not significantly different from the control group (*P* = 0.49).

## Discussion

The objective of the present study was to determine juvenile Chinook salmon physiological and biochemical shifts associated with typical pathogen infections in the San Francisco Bay Delta. Two common pathogens (*C. shasta* and *P. minibicornis*) were ubiquitous among fish deployed in river based on DNA pathogen screening assays. Significant transcriptomic and biochemical differences were measured between deployed fish and those maintained in our facility; however, there were few differences among the fish in the hypoxia and temperature challenge tests.

Out of the 47 infectious agents tested for, two myxozoan parasites, *C. shasta* and *P. minibicornis*, were detected at 100% prevalence in river-deployed fish (RVB and SRH) after a two-week period. Parasite load appeared to be similar between the two locations based on the similar copy numbers detected in fish gills. These two pathogens are frequently detected in salmonids in California rivers, especially during the time the fish were deployed (spring), when water temperatures are elevated (Stocking *et al.,* 2006; Foott *et al.,* 2007; Voss *et al.,* 2018; Lehman *et al.,* 2020). *Ceratonova shasta* and *P. minibicornis* share both vertebrate and invertebrate hosts (Bartholomew *et al.,* 1997; Stone *et al.,* 2008). Their life cycles include an invertebrate polychaete host—*Manayunkia sp*., which when infected releases the actinospore stage into the water column, which can subsequently infect the vertebrate salmonid host. The actinospore then develops within the vertebrate host: salmon or trout species, into a myxospore. Once released from an infected fish, the myxospore infects the polychaete host to complete the life cycle (Bartholomew *et al.,* 1997). Because *C. shasta* and *P. minibicornis* share the same invertebrate host there is the potential for a fish to be simultaneously exposed to actinospores from both parasites (Voss *et al.,* 2018).

*Ceratonova shasta* is the etiological agent of the myxozoan-associated enteronecrosis and has been regarded as the major cause of mortality in juvenile salmonids that migrate through the Pacific Northwest of North America (Foott *et al.,* 2002, 2017; Fujiwara *et al.,* 2011). Clinical disease signs include lethargy, darkening of the body surface, abdominal distension and haemorrhaging in the area of the vent (Conrad and Decew, 1966; Bartholomew *et al.,* 1989). *Parvicapsula minibicornis* was first discovered infecting kidneys of sockeye salmon in an isolated population in Weaver Creek, British Columbia, in 1995 (Kent *et al.,* 1997) and has since been found infecting salmonids in several other river systems along the Pacific Northwest (Foott *et al.,* 2007). In severe infections of the kidney, glomerulonephritis, characterized by diffuse thickening of the basement membrane, occlusion of the glomerular capillaries and necrosis of the tubular epithelium occurs (Raverty *et al.,* 2000). This disease is suspected to induce pre-spawn mortality; however, information regarding salmon susceptibility to this pathogen, especially for juvenile stages, is scarce (St-Hilaire *et al.,* 2002; Bradford *et al.,* 2010). None of these clinical disease signs were observed when fish were visually inspected at the end of the caging period; however, the exposure duration may not have been sufficient to observe them.

Despite being similarly infected by two myxozoans parasites, fish deployed at both river sites displayed contrasting patterns of gene expression, suggesting that environmental factors other than, or in addition to, *C. shasta* and *P. minibicornis* infections likely impacted transcriptomic responses. In the RVB group, out of 18 tested genes involved in the fish immune system and 7 genes involved in the general stress response, 13 genes were differentially expressed, including the upregulation of genes associated with the innate and adaptive immune responses, as well as more and general stress responses. In contrast, in fish deployed at SRH, only *serum amyloid protein A* (*SAA*), a major acute-phase reactant and an effector of innate immunity, was positively regulated compared to the control condition in response to the pathogen infection. Effluent from the wastewater treatment plant located upstream from SRH did not seemingly elicit a response in the individuals tested; however, this study was not geared towards evaluating toxicological impacts. Future studies should include analytical chemistry for contaminants detection in association with pathogen studies. Regulation of these genes was not seemingly associated with pathogen load, suggesting that fish from the RVB group experienced additional forms of stress such as elevated temperatures observed as well as non-targeted pathogens, and xenobiotics; or a potential interactive effect between infection and other environmental parameters. Although our study examined 47 pathogens, it was not inclusive of all possible viruses or intracellular pathogens, therefore it is possible that the fish were infected by other viruses or pathogens not targeted in this study. This hypothesis is supported by the observed up-regulation of MX protein in the RVB group, a gene coding for antiviral proteins and considered as a specific marker of viral infection (Zav’yalov *et al.,* 2019). Similarly, the observed up-regulation of *Cyp450*, *GST3*, *GSTα* and *RHCG*, which are involved in the process of detoxification and are widely used as biomarkers of pollution, further suggests that fish from RVB might have been exposed to a xenobiotics during the 2-week river deployment period (Goksøyr and Förlin, 1992; Lenártová *et al.,* 1997; Nawata *et al.,* 2010). While some of these genes respond to environmental conditions, such as elevated salinity, we did not observe salinity of >1 PSU during the caging period (Boutet *et al.,* 2006; Zanette *et al.,* 2011; Houde *et al.,* 2019). Even though temperatures at RVB were well within Chinook salmon’s range of tolerance (Poletto *et al.,* 2017), fish from this group experienced higher temperatures than those from the SRH and CONT groups (up to 18.7°C; Fig 2) and greater temperature fluctuations (up to 2.5°C) compatible with thermal stress induction (Gallant *et al.,* 2017). This was supported by the differential expression of *HSP90α*, a gene that codes for a chaperone protein that promotes proteins stabilization against heat stress, which is considered as a reliable biomarker of thermal stress (Akbarzadeh *et al.,* 2018).

While it is unclear if stress responses observed following the river deployment period were a consequence of infection, energy reserves and body mass were reduced in river-deployed fish. Liver and heart biochemical analyses conducted on samples collected at the end of the field exposures revealed that while free glucose concentration in the liver and activity of enzymes in the heart were not affected by the deployment, hepatic glycogen concentration was significantly lower in infected, river-deployed fish compared to those in the control group at the end of the 14-day exposure period. Interestingly, while the hepatic glycogen concentration measured in the control group was within the lower range of what has been observed in wild-caught migrating salmonids (10–220 μmol/g of wet liver; French *et al.,* 1983; Dickhoff *et al.,* 1997), river-deployed fish presented depleted hepatic glycogen reserves. Glycogen is the main reserve source of energy for animals and the liver concentration is often associated with the health condition and stress of the fish (Hori *et al.,* 2006; Saravanan *et al.,* 2011). In response to stress, catecholamines stimulate liver glycogenolysis, the conversion of glycogen stored in hepatocytes to free glucose such that it can be circulated by the blood to tissues that need to make ATP (Nakano and Tomlinson, 1967; Andersen *et al.,* 1991; Heath, 1991). The measured reduction in hepatic glycogen concentration likely suggests that environmental conditions in the field were sub-optimal, resulting in a generalized stress response. This hypothesis is supported by the negative growth observed in the fish measured at the end of the field period (i.e. −29 ± 10% and −21 ± 5% of initial body mass in the RVB and SRH groups, respectively). In a natural environment, this stress response could be caused by multiple environmental factors, including the presence of pathogens. The influence of the transition of caged fish feeding from commercial pellets to natural prey cannot be ruled out; however, during the dissection process, the fish were observed to have consumed macroinvertebrates. The measured decrease in body mass and energy reserve can have detrimental consequences on fish survival. Smoltification, and its associated physiological adaptions that allow salmon to survive and grow in seawater, requires the depletion of large stocks of glycogen (Sheridan, 1989). Depleted glycogen stores are likely to delay individuals’ smoltification and outmigration, substantially increasing the risk of predation by a suite of piscivorous fishes and avian predators (Beamesderfer *et al.*, 1996; Hostetter *et al*. 2012; Osterback *et al*. 2013). Furthermore, salmon survival during their first months in the ocean is strongly associated with their size (Claiborne *et al.,* 2011; Duffy and Beauchamp, 2011; Woodson *et al.,* 2013). Larger individuals are also usually less vulnerable to predation, starvation, and extreme environmental conditions (Peterson and Wroblewski, 1984; Sogard, 1997). The capacity to cope with prolonged hypoxia, elevated temperature or sustained swimming activity is lower in smaller individuals because of a limited capacity to rely on anaerobic ATP production (glycolysis) for survival (Johnston and Goldspink, 1973; Nilsson and Östlund-Nilsson, 2008; Corey *et al.,* 2017).

Despite the infection by two pathogens, fish deployed at both river sites did not show detrimental changes in acute hypoxia and thermal stress tolerance. Hypoxia and upper temperature challenge tests conducted 1 and 2 weeks after deployment, respectively, did not reveal any reduced ability in river-exposed groups to contend with rapid environmental change relative to the controls. Fish deployed at RVB, in fact, displayed a higher hypoxia tolerance than the control group. This increased hypoxia tolerance can be the explained by two co-occurring factors. First, several genes involved in general stress response were differentially regulated in fish deployed at RVB. It is therefore possible that stressful environmental conditions at RVB led to changes in the generalized stress response, which might have primed these fish for an additional stressor like hypoxia. For instance, the gene coding for *HSP90a* that was found up-regulated in our study, is also known to be essential to the activation of the hypoxia-inducible factor-1alpha (*HIF-1α*), a key regulator for the physiological response to low oxygen availability (Minet *et al.,* 1999). Second, the magnitude of daily temperature fluctuation observed at RVB combined with the high flows that these fish were exposed to during the caging period, potentially causing the fish to swim more continuously. The forced exercise could have induced phenotypic changes and remodelling, such as increased aerobic potentials in red and white muscles, improved heart performance and better blood oxygen-carrying capacity, which has been shown to lead to an overall increase of the cardiorespiratory performance (Davison, 1997), leading to improved hypoxia tolerance.

In conclusion, we report that all the fish deployed in the Sacramento River were infected with *C. shasta* and *P. minibicornis* over a 2-week period, thus these myxozoan parasites are likely widespread in the Sacramento–San Joaquin River Delta. Field deployment was associated with pathogen detection, a decrease in body mass, reduced glycogen concentration and a transcriptomic response; however, no impacts were observed on stress tolerance as determined through acute hypoxia and upper temperature tolerance, at 1 and 2 weeks post-retrieval of fish, respectively. The determined reduced body mass and liver glycogen content on deployed fish could have been driven by interactions between environmental conditions and caging in the field, besides pathogen infection, highlighting the difficulty in establishing a causal relationship in field tests. Observed reduced mass and hepatic glycogen reserve could have detrimental consequences on juvenile Chinook salmon outmigrating success and overall population stock.

## Funding

This research was supported by the Delta Water Quality and Ecosystem Restoration Grant Program from the California Department of Fish and Wildlife (Proposition 1 grant #P1696002 to R.E.C. and N.A.F.; and grant #P1896051 to R.E.C., N.A.F., A.S., M.B.), the Department of Interior Bureau of Reclamation (grant # R15AC00043 R.E.C.) and the University of California Agricultural Experiment Station Grant (CA-D-ASC-2098-H to N.A.F. and CA-D-ASC-2252-H, CA-D-ASC-2253-RR to A.E.T.).

## Data Availability Statement

The data presented in this study are available on request from the corresponding author.

## Supplementary Material

supplementary_coab102
