# Supplementary material for: Understanding risks and consequences of pathogen infections on the physiological performance of outmigrating Chinook salmon
Source: Conserv Physiol. 2022 Jan 21;10(1):coab102. doi: 10.1093/conphys/coab102 (PMC9040276; doi:10.1093/conphys/coab102)
Supplement: supplementary_coab102 [file supplementary_coab102.zip › S1.docx]

| Agent type | Species | Abbreviation | Forward Primer Sequence (5'-3') | Reverse Primer Sequence (5'-3') | Probe Sequence (FAM-5'-3'-MGB) |
| --- | --- | --- | --- | --- | --- |
| Bacterium | *Aeromonas hydrophila* | ae_hyd | ACCGCTGCTCATTACTCTGATG | CCAACCCAGACGGGAAGAA | TGATGGTGAGCTGGTTG |
| Bacterium | *Aeromonas salmonicida* | ae_sal | TAAAGCACTGTCTGTTACC | GCTACTTCACCCTGATTGG | ACATCAGCAGGCTTCAGAGTCACTG |
| Virus | *Atlantic salmon paramyxovirus* | aspv | CCCATATTAGCAAATGAGCTCTATCTT | CGTTAAGGAACTCATCATTGAGCTT | AGCCCTTTTGTTCTGC |
| Bacterium | *Candidatus Branchiomonas cysticola* | c_b_cys | AATACATCGGAACGTGTCTAGTG | GCCATCAGCCGCTCATGTG | CTCGGTCCCAGGCTTTCCTCTCCCA |
| Parasite | *Ceratomyxa shasta* | ce_sha | CCAGCTTGAGATTAGCTCGGTAA | CCCCGGAACCCGAAAG | CGAGCCAAGTTGGTCTCTCCGTGAAAAC |
| Parasite | *Cryptobia salmositica* | cr_sal | TCAGTGCCTTTCAGGACATC | GAGGCATCCACTCCAATAGAC | AGGAGGACATGGCAGCCTTTGTAT |
| Parasite | *Dermocystidium salmonis* | de_sal | CAGCCAATCCTTTCGCTTCT | GACGGACGCACACCACAGT | AAGCGGCGTGTGCC |
| Parasite | *Facilispora margolisi* | fa_mar | AGGAAGGAGCACGCAAGAAC | CGCGTGCAGCCCAGTAC | TCAGTGATGCCCTCAGA |
| Bacterium | *Flavobacterium psychrophilum* | fl_psy | GATCCTTATTCTCACAGTACCGTCAA | TGTAAACTGCTTTTGCACAGGAA | AAACACTCGGTCGTGACC |
| Virus | *Flavivirus salmonis* | fl_sal | AGCCCTCGCAGGTTCTTTC | GTTGCCCCATTGCACCAT | CCCGCAACCTTGAC |
| Parasite | *Gyrodactylus salaris* | gy_sal | CGATCGTCACTCGGAATCG | GGTGGCGCACCTATTCTACA | TCTTATTAACCAGTTCTGC |
| Parasite | *Ichthyophonus hoferi* | ic_hof | GTCTGTACTGGTACGGCAGTTTC | TCCCGAACTCAGTAGACACTCAA | TAAGAGCACCCACTGCCTTCGAGAAGA |
| Parasite | *Ichthyophthirius multifiliis* | ic_mul | AAATGGGCATACGTTTGCAAA | AACCTGCCTGAAACACTCTAATTTTT | ACTCGGCCTTCACTGGTTCGACTTGG |
| Virus | *Infectious hematopoietic necrosis virus* | ihnv | AGAGCCAAGGCACTGTGCG | TTCTTTGCGGCTTGGTTGA | TGAGACTGAGCGGGACA |
| Virus | *Infectious salmon anemia virus* | isav8 | TGGGCAATGGTGTATGGTATGA | GAAGTCGATGAACTGCAGCGA | CAGGATGCAGATGTATGC |
| Parasite | *Kudoa thyrsites* | ku_thy | TGGCGGCCAAATCTAGGTT | GACCGCACACAAGAAGTTAATCC | TATCGCGAGAGCCGC |
| Parasite | *Loma Salmonae* | lo_sal | GGAGTCGCAGCGAAGATAGC | CTTTTCCTCCCTTTACTCATATGCTT | TGCCTGAAATCACGAGAGTGAGACTACCC |
| Bacterium | *Moritella viscosa* | mo_vis | CGTTGCGAATGCAGAGGT | AGGCATTGCTTGCTGGTTA | TGCAGGCAAGCCAACTTCGACA |
| Parasite | *Myxobolus articus* | my_arc | TGGTAGATACTGAATATCCGGGTTT | AACTGCGCGGTCAAAGTTG | CGTTGATTGTGAGGTTGG |
| Parasite | *Myxobolus cerebralis* | my_cer | GCCATTGAATTTGACTTTGGATTA | ACCATTCATGTAAGCCCGAACT | TCGAAGCCTTGACCATCTTTTGGCC |
| Parasite | *Myxobolus insidiosus* | my_ins | CCAATTTGGGAGCGTCAAA | CGATCGGCAAAGTTATCTAGATTCA | CTCTCAAGGCATTTAT |
| Fluke | *Nanophyetus salmincola* | na_sal | GATCTGCATTTGGTTCTGTAACA | CCAACGCCACAATGATAGCTATAC | TGAGGCGTGTTTTATG |
| Parasite | *Neoparamoeba perurans* | ne_per | GTTCTTTCGGGAGCTGGGAG | GAACTATCGCCGGCACAAAAG | CAATGCCATTCTTTTCGGA |
| Parasite | *Nucleospora salmonis* | nu_sal | GCCGCAGATCATTACTAAAAACCT | CGATCGCCGCATCTAAACA | CCCCGCGCATCCAGAAATACGC |
| Virus | *Salmonid herpesvirus / Oncorhynchus masou herpes virus* | omv | GCCTGGACCACAATCTCAATG | CGAGACAGTGTGGCAAGACAAC | CCAACAGGATGGTCATTA |
| Parasite | *Parvicapsula kabatai* | pa_kab | CGACCATCTGCACGGTACTG | ACACCACAACTCTGCCTTCCA | CTTCGGGTAGGTCCGG |
| Parasite | *Parvicapsula minibicornis* | pa_min | AATAGTTGTTTGTCGTGCACTCTGT | CCGATAGGCTATCCAGTACCTAGTAAG | TGTCCACCTAGTAAGGC |
| Parasite | *Parvicapsula pseudobranchicola* | pa_pse | CAGCTCCAGTAGTGTATTTCA | TTGAGCACTCTGCTTTATTCAA | CGTATTGCTGTCTTTGACATGCAGT |
| Parasite | *Paranucleospora theridion (syn. Desmozoon lepeophtherii)* | pa_ther | CGGACAGGGAGCATGGTATAG | GGTCCAGGTTGGGTCTTGAG | TTGGCGAAGAATGAAA |
| Bacterium | *Piscichlamydia salmonis* | pch_sal | TCACCCCCAGGCTGCTT | GAATTCCATTTCCCCCTCTTG | CAAAACTGCTAGACTAGAGT |
| Bacterium | *Piscirickettsia salmonis* | pisck_sal | TCTGGGAAGTGTGGCGATAGA | TCCCGACCTACTCTTGTTTCATC | TGATAGCCCCGTACACGAAACGGCATA |
| Virus | *Piscine myocarditis virus* | pmcv1 | AGGGAACAGGAGGAAGCAGAA | CGTAATCCGACATCATTTTGTGA | TGGTGGAGCGTTCAA |
| Virus | *Piscine reovirus* | prv | TGCTAACACTCCAGGAGTCATTG | TGAATCCGCTGCAGATGAGTA | CGCCGGTAGCTCT |
| Bacterium | *Renibacterium salmoninarum* | re_sal | CAACAGGGTGGTTATTCTGCTTTC | CTATAAGAGCCACCAGCTGCAA | CTCCAGCGCCGCAGGAGGAC |
| Bacterium | *Rickettsia-like organism* | rlo | GGCTCAACCCAAGAACTGCTT | GTGCAACAGCGTCAGTGACT | CCCAGATAACCGCCTTCGCCTCCG |
| Virus | *Salmon alphavirus 1, 2, and 3* | sav | CCGGCCCTGAACCAGTT | GTAGCCAAGTGGGAGAAAGCT | TCGAAGTGGTGGCCAG |
| Bacterium | *Gill chlamydia* | sch | GGGTAGCCCGATATCTTCAAAGT | CCCATGAGCCGCTCTCTCT | TCCTTCGGGACCTTAC |
| Virus | *Infectious salmon anemia virus* | snow7 | CAGGGTTGTATCCATGGTTGAAATG | GTCCAGCCCTAAGCTCAACTC | CTCTCTCATTGTGATCCC |
| Parasite | *Sphaerothecum destructuens* | sp_des | GGGTATCCTTCCTCTCGAAATTG | CCCAAACTCGACGCACACT | CGTGTGCGCTTAAT |
| Parasite | *Spironucleus salmonicida* | sp_sal | GCAGCCGCGGTAATTCC | CGAACTTTTTAACTGCAGCAACA | ACACGGAGAGTATTCT |
| Parasite | *Tetracapsuloides bryosalmonae* | te_bry | GCGAGATTTGTTGCATTTAAAAAG | GCACATGCAGTGTCCAATCG | CAAAATTGTGGAACCGTCCGACTACGA |
| Bacterium | *Tenacibaculum maritimum* | te_mar | TGCCTTCTACAGAGGGATAGCC | CTATCGTTGCCATGGTAAGCCG | CACTTTGGAATGGCATCG |
| Virus | *Viral erythrocytic necrosis virus* | ven | CGTAGGGCCCCAATAGTTTCT | GGAGGAAATGCAGACAAGATTTG | TCTTGCCGTTATTTCCAGCACCCG |
| Virus | *Viral encephalopathy and retinopathy virus* | ver | TTCCAGCGATACGCTGTTGA | CACCGCCCGTGTTTGC | AAATTCAGCCAATGTGCCCC |
| Bacterium | *Vibrio anguillarum* | vi_ang | CCGTCATGCTATCTAGAGATGTATTTGA | CCATACGCAGCCAAAAATCA | TCATTTCGACGAGCGTCTTGTTCAGC |
| Bacterium | *Vibrio salmonicida* | vi_sal | GTGTGATGACCGTTCCATATTT | GCTATTGTCATCACTCTGTTTCTT | TCGCTTCATGTTGTGTAATTAGGAGCGA |
| Bacterium | *Yersinia ruckeri* | ye_ruc | TCCAGCACCAAATACGAAGG | ACATGGCAGAACGCAGAT | AAGGCGGTTACTTCCCGGTTCCC |

Supplemental Data S1: Summary of the 47 infectious agents of interest, their corresponding abbreviation, primers and probes sequences.
