# Supplementary material for: Understanding risks and consequences of pathogen infections on the physiological performance of outmigrating Chinook salmon
Source: Conserv Physiol. 2022 Jan 21;10(1):coab102. doi: 10.1093/conphys/coab102 (PMC9040276; doi:10.1093/conphys/coab102)
Supplement: supplementary_coab102 [file supplementary_coab102.zip › S2.docx]

| Gene Name | Abbreviation | Function | Forward Primer Sequence (5'-3') | Reverse Primer Sequence (5'-3') | Roche UPL Probe number |
| --- | --- | --- | --- | --- | --- |
| Cytochrome P450 Family 1 Subfamily A Member 1 | CYP450 | General Stress | GCTGTCCCGTAGACTGACTGCT | CGCCCACCATTGACTACACC | 165 |
| Glutathione-s-Transferase 3 | GST3 | General Stress | ACGGGAATGTAACGGCTTGTA | TCACCTGACGCCAAGAAAACT | 65 |
| Glutathione-s-Transferase alpha | GSTα | General Stress | CCACCATTGCCTACTCCTTCC | TGCTCTCAGTTTGGGGTAACG | 26 |
| Heat Shock protein serpin H1 | HSP47 | General Stress | CCAGCATCTTCGGAAGTGAG | AAGGGATGGTCAGCGTAGAA | 73 |
| Heat Shock Protein 90 kDa AA1-inducible form | HSP90α | General Stress | ATACCACCCCGGCTTCAG | TCTTGAGTGACTTTGCTGACAGA | 22 |
| Heat Shock Protein 90 kDa alpha Beta 1 | HSP90αβ | General Stress | AGAGGAGGAGGCTGAGACCTTT | TGATGATCAGGGACATGAGCTG | 13 |
| Ammonium transporter | RHCG | General Stress | CAGGTAGGTGTTGATGGCTGC | CTAGTTTTAACTCGGCCATCTCG | 44 |
| Glyceraldehyde-3-Phosphate Dehydrogenase | GAPDH | Housekeeping Gene | CAGAAAGCTGGAATGGGACT | GTGGAGGGCTGGGGTACTA | 47 |
| 60S Ribosomal gene 7l | RPL7 | Housekeeping Gene | GCAAGGTCGGGAACTTCTACG | CCCCTGATCCTGATGACGAA | 117 |
| Ribosomal Protein S9 | RPS9 | Housekeeping Gene | TTGTCGCAAGACATACGTCAC | TCCTGGTCCAGACGAGACTT | 70 |
| Beta-2-Microglobulin | B2M | Immune System | ATACCTCTGGCACTTTAGTCAGACTG | GTAATCCAAAAGGCACAGACACAC | 21 |
| C-type Lysozyme | C-Lys | Immune System | GATCGCCACTGTGAGGTCATC | GGGGCTAAGAACGTCTGTGGT | 164 |
| Complement factor CF3 | CF3 | Immune System | CCTGGGCAAGATGGGTGA | AGAAGCTGCGGAACTCATCC | 24 |
| Complement factor BF-2 | CFB | Immune System | ATCAGCAGCGCTACACACA | GGACCTGTGATTCACCATGA | 67 |
| Cold inducible RNA Binding Protein | CIRBP | Immune System | GGCAGCGGAGGTTACAGAGAG | CCAGAGCGGCCACCATAAC | 133 |
| Chemokine Receptors 5 | CR5 | Immune System | GGGAACGTTCTGGTCCTGT | GTCGGTCATGTTGCGTAGTTT | 3 |
| Chemokine Receptors 6 | CR6 | Immune System | AACTTCAGGTTCCCAGACAACG | CGTAGCAGAAGCCCATGACC | 4 |
| Chemokine Receptors 7 | CR7 | Immune System | TGGTTCATGCCCACCTTCTAC | CACCAGGATGTTACCGACCAG | 38 |
| Classical Immunoglobulin | IgM | Immune System | CAGCTGCCTCCTGTGTTCACT | GACCTGGCTGGACAGTCATAGAG | 102 |
| Chemokine Interleukin 1 beta | IL-1β | Immune System | GACACATGTCCACCGGTTTG | CCTGGAGTCTGCCAGGTTCA | 18 |
| Chemokine Interleukin 8 | IL-8 | Immune System | GCGATGTCGCTGCATTGA | CGAGCTGGGAGGGAACATC | 108 |
| Major Histocompatibility complex II | MHC2 | Immune System | CAATGCAAAGAGTGGATAAATGA | TGAGCCTAGAGAAGCAGGTGA | 62 |
| MX protein | MXpro | Immune System | CCACTGCCAACCATTGCTG | CCCAGAAAGGGTCTCAAGCAC | 54 |
| Serum amyloid protein A | SAA | Immune System | CTCGGGGCAACTATGATGCT | CATCTCCCGGCCATTACTGAT | 44 |
| T-cell receptor alpha | TCRα | Immune System | AAACCAGAATTCCTGCTCCTCAT | CGAGGGTGGGTCGATTCAT | 134 |
| T-cell receptor beta | TCRβ | Immune System | CGAGGGTTTGTTTGAGGAGA | CGAGAGTTCCCTTCAGGTTTC | 29 |
| Toll like receptors 1 | TLR1 | Immune System | TGGAGGGGATCATGTACTGAGG | GAGGGTCTGACAGTGTGATCCTG | 1 |
| Toll like receptors 2 | TLR2 | Immune System | CAGCTGGTCCAACTGTCTGACTC | TCTCTGACCAGGCTGAGGTTTC | 55 |
| Toll like receptors 3 | TLR3 | Immune System | TTGGGCCAGCTACAGGAGAT | GAACGACATAGGGCTGAGGATG | 164 |
| Tumor necrosis factor alpha | TNF-α | Immune System | CCCTGTGTACCCAGCAACG | TGCAGCTGAGACACACAATGC | 7 |

Supplemental Data S2: List of the 25 candidate genes of interest plus 3 housekeeping genes, their corresponding abbreviation, overall function, primer sequences and Roche probe number used.
