# Supplementary material for: Understanding risks and consequences of pathogen infections on the physiological performance of outmigrating Chinook salmon
Source: Conserv Physiol. 2022 Jan 21;10(1):coab102. doi: 10.1093/conphys/coab102 (PMC9040276; doi:10.1093/conphys/coab102)
Supplement: supplementary_coab102 [file supplementary_coab102.zip › S3.docx]

Supplemental Data S3: Heatmap of the detection of 47 infectious agents in gills from fish sampled 1 day before and immediately after the field deployment. CONT: fish from the control group; RVB: fish deployed in the Sacramento River at Rio Vista Bridge, and SRH: fish deployed in the Sacramento River at Hood. Black indicates absence of pathogen detection and red intensity corresponds to the logarithmic concentration of mRNA absolute copy number / µl, as an indication of overall abundance. Pathogen detection by means of cDNA indicates live organisms at the time of sampling.
